# TaxKB: a knowledge base for new taxane-related drug discovery

**DOI:** 10.1186/s13040-015-0053-5

**Published:** 2015-06-28

**Authors:** Kasi Murugan, Sangeetha Shanmugasamy, Saleh Al-Sohaibani, Naga Vignesh, Kandavel Palanikannan, Antonydhason Vimala, Gopal Ramesh Kumar

**Affiliations:** 1 Department of Botany and Microbiology, College of Science, King Saud University, P.O. Box 2455, Riyadh, 11451 Saudi Arabia; 2 Bioinformatics Lab, AU-KBC Research Centre, MIT Campus, Anna University, Chennai, 600 044 Tamil Nadu India

**Keywords:** Anticancer drugs, Database, Drug discovery, Taxanes, Semisynthetic taxanes

## Abstract

**Background:**

Taxanes are naturally occurring compounds which belong to a powerful group of chemotherapeutic drugs with anticancer properties. Their current use, clinical efficacy, and unique mechanism of action indicate their potentiality for cancer drug discovery and development thereby promising to reduce the high economy associated with cancer worldwide. Extensive research has been carried out on taxanes with the aim to combat issues of drug resistance, side effects, limited natural supply, and also to increase the therapeutic index of these molecules. These efforts have led to the isolation of many naturally occurring compounds belonging to this family (more than 350 different kinds), and the synthesis of semisynthetic analogs of the naturally existing molecules (>500), and has also led to the characterization of many (>1000) of them. A web-based database system on clinically exploitable taxanes, providing a link between the structure and the pharmacological property of these molecules could help to reduce the druggability gap for these molecules.

**Results:**

Taxane knowledge base (TaxKB, http://bioinfo.au-kbc.org.in/taxane/Taxkb/), is an online multi-tier relational database that currently holds data on 42 parameters of 250 natural and 503 semisynthetic analogs of taxanes. This database provides researchers with much-needed information necessary for drug development. TaxKB enables the user to search data on the structure, drug-likeness, and physicochemical properties of both natural and synthetic taxanes with a “General Search” option in addition to a “Parameter Specific Search.” It displays 2D structure and allows the user to download the 3D structure (a PDB file) of taxanes that can be viewed with any molecular visualization tool. The ultimate aim of TaxKB is to provide information on Absorption, Distribution, Metabolism, and Excretion/Toxicity (ADME/T) as well as data on bioavailability and target interaction properties of candidate anticancer taxanes, ahead of expensive clinical trials.

**Conclusion:**

This first web-based single-information portal will play a central role and help researchers to move forward in taxane-based cancer drug research.

## Background

Worldwide, cancer is a major public health issue accounting for approximately 8.2 million deaths and approximately 14 million new cases in 2012 that is expected to rise to 22 million annually within the next 20 years [http://globocan.iarc.fr/Pages/fact_sheets_cancer.aspx]. Globally vast amount of resources are being invested for the prevention, diagnosis, and treatment of cancer. Natural compounds have often offered new leads for novel molecular structures with anticancer activity whose synthesized analogs showed improved efficacy and toxicity profiles [1]. The chemical diversity of these compounds has long been explored as a rich source for the identification of unique scaffold of structures that led to many natural product-based drugs in the market. A recent review reports that in 49 % of all cancer drugs, it was either the natural product or its derivative that served as a starting point for the rational drug design process [2]. Among the recently discovered chemotherapeutic drugs, taxanes are considered to be the most powerful group of compounds [3]. Moreover, some of the promising results from clinical studies have led various oncologists, researchers, and drug manufacturers to anticipate their future consideration and development in the field of cancer [4].

National Cancer Institute discovered the first taxoid in 1960s. Schiff and his coworkers revealed the unique pharmacological mechanisms of taxanes during 1979. These taxanes bind to the microtubules, stabilize them, and prevent their depolymerization thereby inhibiting cell growth [5]. Furthermore, mechanisms of their molecular action are well characterized and include (i) cell division control 2 (cdc-2) kinase activation, (ii) cyclin B-1 stabilization, (iii) spindle assembly checkpoint activation, (iv) apoptosis induction through B-cell lymphoma 2 (bcl-2) phosphorylation, and (v) inhibition of cell proliferation [6]. The natural diterpene taxane, Paclitaxel and the semisynthetic taxane, Docetaxel are deemed to be the most important among the anticancer drugs. Their discovery was a breakthrough which is now considered a major milestone in natural product-based anticancer drug discovery and development [7]. There are several key reasons why taxanes are of importance in cancer drug discovery. These include their clinical efficacy, potential for modification and improvement, unique mechanism of action on tubulin polymerization rather than the DNA, suitability for combination therapy along with novel mitotic target agents being developed, as well as selective antivascular action at nontoxic doses [8]. Furthermore, the development of novel taxane derivatives with enhanced anticancer activity, fewer and milder side effects, and superior pharmacological properties would not only maximize the induced benefits but also will reduce the high economic and human costs worldwide [3].

Unfortunately, these agents suffer from issues such as drug resistance, lack of specificity, toxicity, poor solubility, and limited natural occurrence and availability. The issue of drug resistance leads to a lack of response and eventual relapse of the disease in many patients. Moreover, limited solvent availability for these compounds restricts dose escalation and increases their toxicity which necessitates the use of premedications [9]. They are not cell-specific and do not show similar results on the microtubules when used at different concentrations (they cause microtubule arrangement into bundles at higher concentrations while suppressing and stabilizing microtubule dynamics without altering the polymer mass at lower concentrations) [4]. Therefore, therapeutic index, defined as the ratio of the TD50 (or LD50) to the ED50 as determined from quantal dose–response curves [10] which indicates the safety profile of taxanes, needs to be improved either by developing new drugs that target microtubules at different sites, or by improving drug scheduling or formulations or by increasing drug specificity to increase antitumor activity and decrease its toxicity [9]. Despite some of these disadvantages, the utility of these compounds in cancer cannot be disregarded, given their advantages [4]. For example, it has been found that a suitable modification of toxoids at the C10 site and replacement of phenyl group at C3 position with an alkenyl or alkyl group leads to second-generation toxoids with one to two orders of magnitude more potency against drug-resistant cancer cell lines [11]. Moreover, the scarcity of taxanes from natural sources also impedes their further development. Currently, commercial demands are met through alternate semisynthetic processing of their more abundant precursors. For example, Docetaxel is derived from 10-deacetylbacatin III, a precursor found in more abundant *Taxus* (yew) species [12]. Analogs of existing drugs have also been synthesized to increase drug targeting, pharmacokinetic profile, and drug supply [1]. To overcome the aforementioned clinical problems, the structure of taxanes has been extensively modified to obtain new molecules with a better therapeutic index and the ability to overcome drug resistance [13]. The enormous scope of taxanes encouraged extensive and intensive research which led to the isolation of 350 natural taxanes from yews, synthesis of more than 500 semisynthetic analogs, and characterization of thousands of taxane molecules. The sheer volume of these discoveries motivates the need for a web-based database system to help cancer drug developers in the war against cancer.

Although a number of databases on chemically active natural compounds have been published [3], there is no database devoted to the taxane family of compounds, despite their success as antitumor agents. Examples of these existing databases on natural compounds other than taxanes include SuperNatural [14], CancerResource [15], and NPACT [2]. Hence, a comprehensive web-based database of taxanes linking their chemical structures with known pharmacological properties is a much-needed tool in cancer drug development. Such a tool will improve our understanding of these compounds by allowing easy screening of clinically exploitable taxanes, serving quantitative structure–activity relationships, and helping to optimize the initial lead compounds from them. These improvements promise economic as well as scientific gains for the pharmaceutical industry, thus enabling the creation of this platform.

## Results and discussion

### Taxanes

**Fig. 1:**
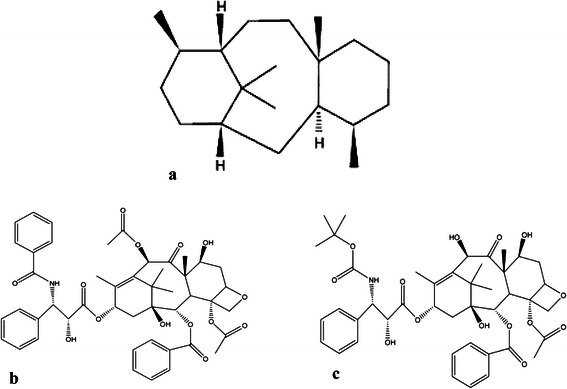
Structures of (**a**) Taxane (**b**) Paclitaxel and (**c**) Docetaxel [5]

Several types of cancers including breast, ovarian, head and neck, lung, and prostate cancer were treated by well-known anticancer drugs that belong to the taxane family. Taxanes are biologically active diterpenes, a large and varied class of hydrocarbons with four isoprene units that are produced from the bark of the yew tree [16]. These biologically active, highly oxygenated, and esterified diterpenes’ unique structure (Fig. 1a) consists of a tetracyclic core frame called baccatin III, with four core rings named ring A (cyclohexene), ring B (cyclooctane), ring C (cyclohexane), and ring D (oxetane). Important research work has been carried out to determine the structural features of taxanes thereby revealing the biological activity of each component of the molecule. These efforts have previously been reviewed [17]. In 1971, Wall and coworkers showed the *in vitro* anticancer activity of Paclitaxel, originally isolated from Pacific Yew (*Taxus brevifolia*), which was later approved for the treatment of breast cancer by the Food and Drug Administration (FDA) in the United States. Further developments of the compound led to the realization of a semisynthetic taxane (Docetaxel) and other taxane analogs. The taxanes, namely, Paclitaxel and Docetaxel, routinely used in clinical practice, are known as classical taxanes [18]. The low concentration of Paclitaxel in the original *T. brevifolia* and the supply crisis of Taxol or Taxotere stirred the interest to produce these compounds by means of isolation from all other 10 geographically isolated *Taxus* species including *T. canadensis*, *T. cuspidate*, *T. mairei*, *T. baccata*, *T. wallichiana*, and *T. yunnanensis*. This effort resulted in the identification of more than 350 taxane diterpenoids from these yew trees, expanding the diterpenoid family, which continues to grow in size [19]. However, direct isolation from natural sources is limited due to the slow growth of yew trees, their limited numbers in nature, as well as very high costs, and strict national forest protection policies [20]. This has consequently sparked interest in active exploration for new taxane drugs using natural taxanes as starting materials for synthesis and its modifications, biotransformation employing *Ginkgo*, and *Platycodon grandiflorus* cell suspension cultures [19]. *Taxus* cell culture is one example of such a renewable method for the production of this molecule. This method has been found to be capable of producing a large amount of 4(20), 11(12)-taxadienes with C-14 oxygenated functional groups rather than with C-13 oxygenated functional groups [21]. Fig. 1 shows the structures of taxane, Paclitaxel and Docetaxel.

### TaxKB

**Fig. 2:**
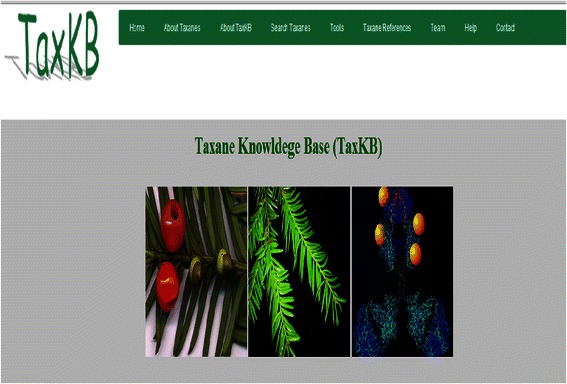
Screenshot of TaxKB home page with key features and search options

TaxKB is an online multi-tier relational database storing drug development information pertaining to taxanes and their analogs. It currently holds 11,000 entries with 42 parameters for each taxane. More than 250 natural taxanes have been documented in TaxKB including the well characterized ones such as Taxol, 10-Deacetylbaccatin III, Cephalomannine, 10-Deacetyl taxol, 2′,7-bis-Acetyl taxol, 10-Deactyl taxol, 10-Deactyl taxol B, 10-Deactyl taxol C, 10-Deactyl-7-xylosyltaxol, Cephalomannine, Taxol C, Xylosyltaxol C, and Xylosyltaxol that show anticancer activity. It also includes a total of 503 semisynthetic taxane analogs collected from the literature. TaxKB enables users to search specifically for individual properties of taxanes with a “General Search” option in addition to a “Parameter Specific Search” option. It displays taxanes’ two-dimensional (2D) as well as three-dimensional (3D) structure that can be downloaded (as a PDB file) and viewed by any molecular visualization tool. A screenshot of TaxKB website’s home page is shown in Fig. 2. At present, nearly 1,000 taxane molecules are characterized and published in several journals.

The current new cancer therapy development approaches are difficult, costly, and time-consuming, potentially taking >10 years to develop a new drug and frequently costs more than 1 billion US dollars. In addition, the level of attrition is very high with about 80–95 % of new drugs failing in the course of clinical trials due to lack of efficacy or unacceptable toxicity [22]. Hence, the ultimate aim of the present TaxKB database was to provide candidate anticancer drug taxanes’ ADME/T (Absorption, Distribution, Metabolism, and Excretion/Toxicity) information ahead of expensive clinical trials for predicting their bioavailability and target interacting properties. If the taxane or its fragments follows the Lipinski’s Rule of five (low molecular weight ≥500, H bond donor ≥5, H bond acceptors ≥10, log P ≥ 5) then the TaxKB information will allow for their optimization into leads [23].

The following pieces of information were retrieved from this database suggesting its usefulness for taxane-based cancer drug research and for mining the most appropriate taxane for clinical development. (i) Taxanes have a minimum molecular weight of 200 Da and the percentage of taxanes weighing between 600 and 800 Da is about 45 %. (ii) All taxanes have a similar composition with carbon present at the highest percentage of about 70 %, oxygen at an average of 20 %, and hydrogen at about 10 %, while in some compounds nitrogen is also present at low numbers (e.g. 10-Deacetyltaxine I, 10-Dideacetyltaxine II, and 9-Deacetyltaxine I). (iii) Overall, 50 % of all taxanes’ monoisotopic mass values are in the range of 600–800. (iv) About 32 % of taxanes have a molar volume that lies in the range of 500–600 Da, within that range, 31 % weigh between 400 and 500 Da. Information such as these provided by the database, will be highly useful for selecting the best taxanes as drug candidates.

## Methods

### Data organization

**Fig. 3:**
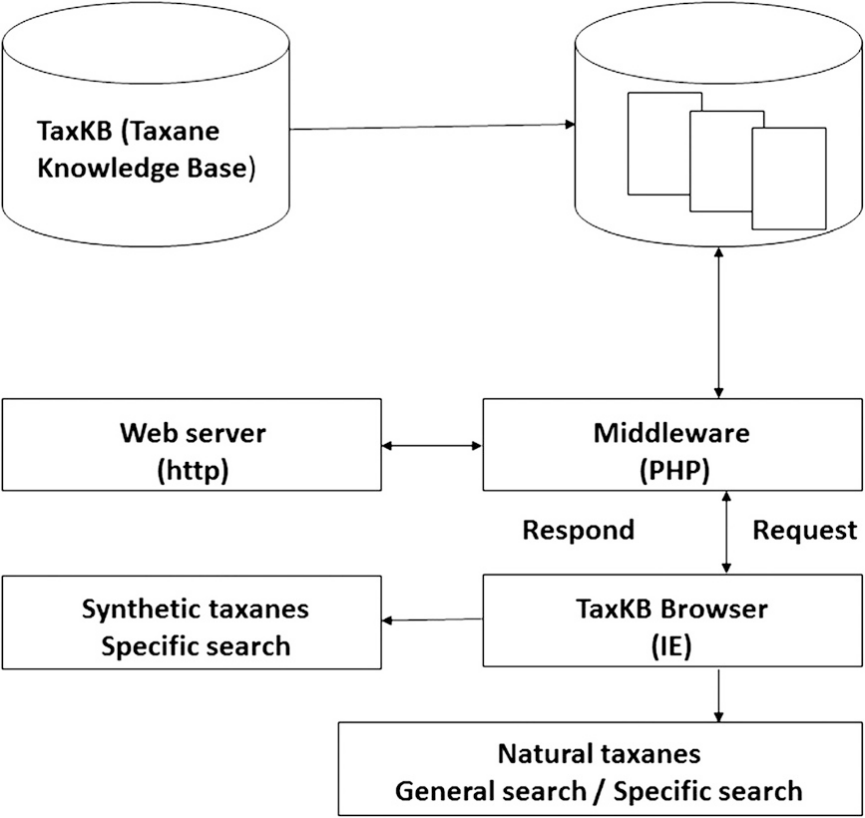
Data flow and three level schema architecture of TaxKB

MySQL Server Edition 5.1 was used for the construction of the TaxKB database. This highly configurable and extensible user-friendly database retrieval interface was developed using PHP integrated with HTML. PubMed database searches were conducted for English language publications using the search terms “taxane” and their derivatives. The knowledge obtained through various resources and data on structures, drug-likeness, and physicochemical properties of taxanes are made available on TaxKB. The structure of taxanes is generated using structure and draw mode of ChemSketch 12.0 (http://www.acdlabs.com) and are used for their property calculation. Marvin (http://www.chemaxon.com) was used to generate 3D structures in PDB format. For the data of physicochemical property, the LogP, molar refractivity, molar volume, surface tension, and polarization are the included curated information. For assessing the compound of interest for drug-likeness, the properties such as hydrogen bond donor’s count, hydrogen bond acceptor’s count, rotatable bond, total polar surface area (TPSA), and toxicity [24] are curated. For the interactive display of the current content, 2D and 3D chemical structures of 259 natural and 503 semisynthetic taxanes were either manually created or downloaded from the PubChem database and Jmol, the java applet-based program is used. The “three level schema architecture” followed in the creation of the database is provided in Fig. 3. Hence, browsing TaxKB data can now be carried out using several different available features: the “Select taxane name” feature with a list of taxanes, the “Physicochemical properties” feature that allows searching through 17 unique properties of the molecules, the “Drug-like properties” feature allowing selection from 16 drug-likeness properties, and the “Reflinks” feature allowing a search through various IDs from different databases. Links to PubChem, DrugBank, PharmGKB, GenBank, Swiss-Prot, and KEGG database were provided which, in turn, supports more specific information on taxanes.

### Data extraction

**Fig. 4:**
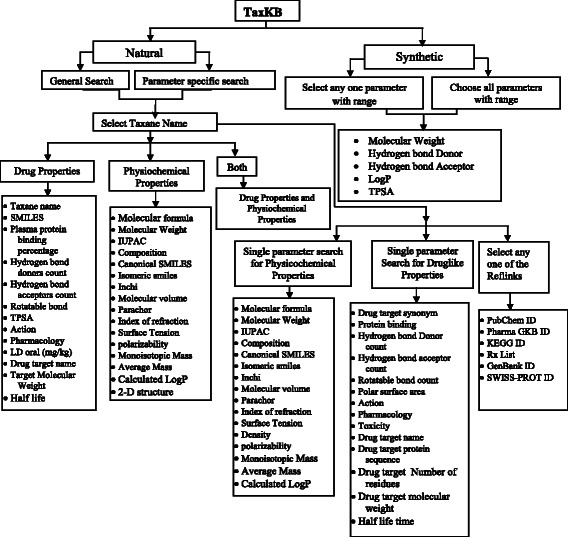
TaxKB Database organization showing the associated information included with each search option

The multi-tier relational database contains all the needed drug development information on taxanes, which can be accessed through its two modes of search: the natural taxane search and synthetic taxane search. Querying the TaxKB database is primarily based on either “General Search” or specific “Parameter Specific Search” (Fig. 4) in the “Search Taxanes’ section of the home page. The general search accepts queries led to retrieve all the properties of particular taxanes in TaxKB. The user can view all the details of the selected taxane by using options such as “Taxane name,” “Drug-like properties,” “Physicochemical properties” or both. Parameter-specific search can be used to access different properties of taxanes. It offers physicochemical properties, drug-like properties, and Reflinks. The parameter-specific search consists of four fields. In the first field, the user can choose any one particular taxane such as Paclitaxel or Taxuspine. In the second field, physicochemical properties (e.g. molecular weight, molecular formula, etc.) can be chosen. From the third field, drug-likeness properties (e.g. H bond donor count, polar surface area, etc.) and similar modus operandi can be applied to the fourth field, that is, “Reflinks” (e.g. PubChem ID and KEGG ID). The user can select a particular property each from the respective field as their requirement and execute the query that displays the desired information. Large availability of data on semisynthetic taxanes makes their retrieval possible by using a simple property-based specific-search. It includes different range of data on molecular weight, hydrogen bond donor, hydrogen bond acceptor, LogP, and TPSA. This method depends upon user’s search criteria with compounds that are retrieved based on either individual property or group of properties.

### Data analysis

The presented web-based TaxKB database is a new resource available for cancer drug developers that would enable easy screening of clinically exploitable taxanes and allow the generation of fragment and scaffold containing libraries and designing of novel taxane leads with properties similar to the original compounds. Key features of the database include the physicochemical and drug-likeness property data of taxanes of both natural and synthetic origin.

## Conclusion

TaxKB is a single-information portal for the most successful blockbuster natural drug compound ever discovered, namely, taxane. To our knowledge, it is the first and only web-based resource integrating multidisciplinary data on taxanes as derived from different drug development efforts so far. It provides a link between two independent processes, taxane-based cancer drug development, and the ADME/T and thus provides a better understanding and earlier detection of potential development failures. Hence, it serves as an excellent online resource for taxane-related drug discovery. The motto of today’s drug discovery and development industry is “Fail faster, Fail cheaper.” As a result, scientists are constantly striving for new ways to eliminate poor drug candidates earlier in the drug discovery cycle. The rapid pace of development of new and advanced taxane analogs can enhance the overall quality of drugs and therefore health care. TaxKB, as an information resource for taxanes, their analogs, and their properties can assist researchers in developing new drug candidates based on the various properties of these molecules. Several enhancements will be carried out in the upcoming release, including incorporation of additional taxane structures as well as more properties of these molecules, as new dimensions that can further improve the present pace of new taxane drug discovery.
